# Learning Autonomous Navigation in Unmapped and Unknown Environments

**DOI:** 10.3390/s24185925

**Published:** 2024-09-12

**Authors:** Naifeng He, Zhong Yang, Chunguang Bu, Xiaoliang Fan, Jiying Wu, Yaoyu Sui, Wenqiang Que

**Affiliations:** 1 College of Automation Engineering, Nanjing University of Aeronautics and Astronautics, Nanjing 211106, China; nfhe@nuaa.edu.cn (N.H.); wujiying@nuaa.edu.cn (J.W.); suiyaoyu@nuaa.edu.cn (Y.S.); quewenqiang@nuaa.edu.cn (W.Q.); 2 State Key Laboratory of Robotics, Shenyang Institute of Automation Chinese Academy of Sciences, Shenyang 110017, China; fanxiaoliang@sia.cn

**Keywords:** mobile robot, mapless, navigation, reinforcement learning

## Abstract

Autonomous decision-making is a hallmark of intelligent mobile robots and an essential element of autonomous navigation. The challenge is to enable mobile robots to complete autonomous navigation tasks in environments with mapless or low-precision maps, relying solely on low-precision sensors. To address this, we have proposed an innovative autonomous navigation algorithm called PEEMEF-DARC. This algorithm consists of three parts: Double Actors Regularized Critics (DARC), a priority-based excellence experience data collection mechanism, and a multi-source experience fusion strategy mechanism. The algorithm is capable of performing autonomous navigation tasks in unmapped and unknown environments without maps or prior knowledge. This algorithm enables autonomous navigation in unmapped and unknown environments without the need for maps or prior knowledge. Our enhanced algorithm improves the agent’s exploration capabilities and utilizes regularization to mitigate the overestimation of state-action values. Additionally, the priority-based excellence experience data collection module and the multi-source experience fusion strategy module significantly reduce training time. Experimental results demonstrate that the proposed method excels in navigating the unmapped and unknown, achieving effective navigation without relying on maps or precise localization.

## 1. Introduction

In unmapped and unknown environments, safe and effective autonomous decision-making and obstacle avoidance present significant challenges for the autonomous navigation of mobile robots. To address these challenges, the three main components of mobile robots—perception, localization, and decision-making—employ various effective methods to solve this problem. In particular, the unmapped environment, though structured, introduces unique difficulties in localization and navigation due to its narrow and monotonous layout, making it a complex environment for testing the robustness of autonomous systems. Autonomous decision-making in such an environment is a significant challenge for the safe and effective navigation of mobile robots. For mobile robots navigating indoor environments, Simultaneous Localization and Mapping (SLAM) [1,2,3] is an essential component of an autonomous navigation system. The generated map enables precise robot localization, facilitating path planning and trajectory tracking applications. However, SLAM has the following limitations: (1) high computational resource demand for data processing and high-precision mapping; (2) sensor accuracy impacting mapping quality; (3). reduced map accuracy in sparse or non-structured environments; and (4) limited navigational accuracy over long distances and in unknown environments. Furthermore, when using low-cost 2D LiDAR sensors (TurtleBot3 LDS-01) in unmapped environments, challenges such as localization errors and multipath reflections can further complicate navigation.

To address these challenges, we utilize artificial intelligence technology to integrate data from low-cost, low-precision ranging sensors into the control system, enabling autonomous robotic decisions even in constrained environments. We employ a data-driven, end-to-end training approach for the agent to accomplish navigation tasks independent of high-precision maps. While there has been significant research on mapless navigation, existing algorithms that integrate traditional methods with deep learning still have limitations in mobile robot navigation [4]. Recently, deep reinforcement learning (DRL) has demonstrated considerable potential in various domains [5,6,7,8], notably in autonomous navigation, motion control, and pattern recognition for mobile robots. However, in unfamiliar and complex environments, the absence of map data can cause robots to converge on local optima, leading to task failures. Additionally, discrepancies between simulation and real environments can render trained strategies ineffective in practice. Visual navigation, although powerful, is sensitive to environmental changes, involves high computational complexity, and has limited generalizability, making the transition from simulation to real-world applications challenging.

**Figure 1:**
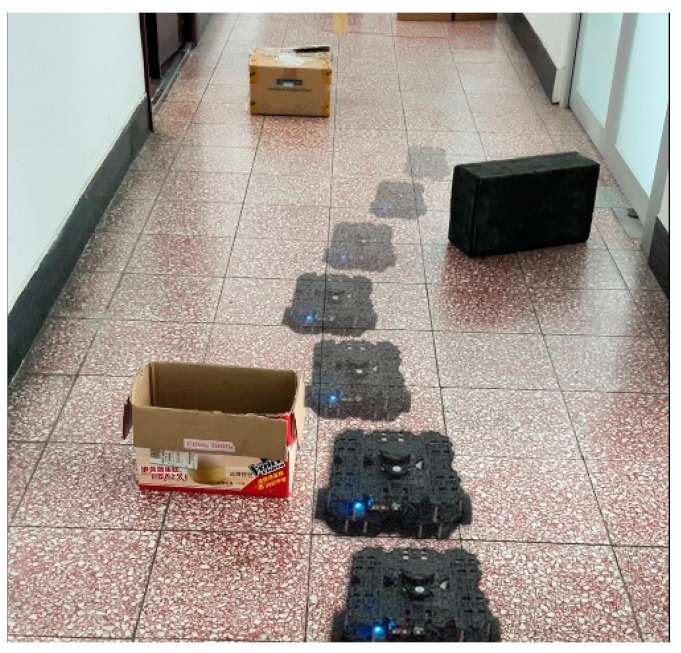
Robot navigates through an Unknown Environment.

**Figure 2:**
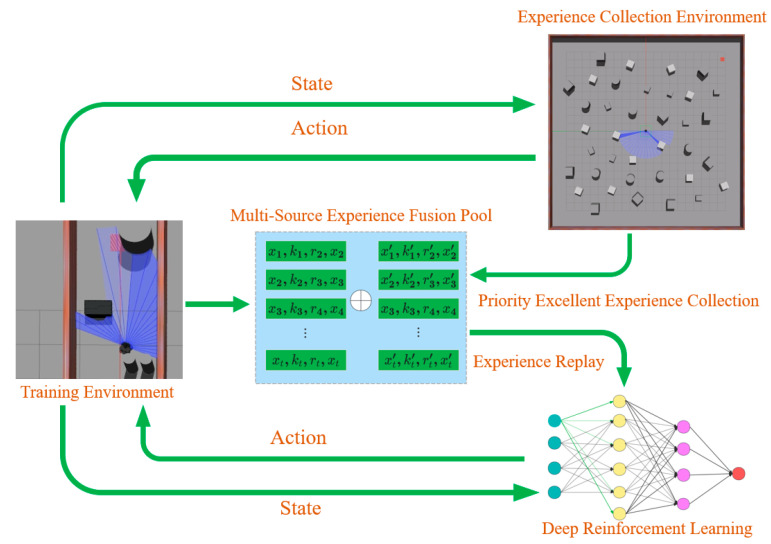
Proposed algorithmic structural framework for autonomous navigation and autonomous obstacle avoidance.

In light of this, we propose a DRL algorithm for unmapped and unknown environments, as illustrated in Figure 1. This study validates the algorithm using the mobile robot “Turtlebot3”, which is equipped solely with a low-cost 2-D LiDAR in both simulation and real-world environments. Despite the lower map accuracy and significant localization errors inherent in such sensors, our approach offers increased versatility and robustness. This algorithm facilitates the completion of navigation tasks via real-time, effective, autonomous decision-making in unknown environments. The structure of the proposed algorithm is detailed in Figure 2. The figure illustrates the entire process, from data collection and prioritization to policy training. Initially, the robot generates initial strategies using the 2D lidar and DRL algorithm, interacting with the environment to produce experience data. The data are then stored and fed into the “Multi-source Experience Fusion Pool”, where they are prioritized based on reward values. Within the “Experience Collection Environment”, the data undergo further prioritization and categorization, contributing to the pool for subsequent strategy optimization. The DRL module continuously refines the strategy through experience replay, enhancing the robot’s performance in complex tasks. The contributions of this paper are in the following three aspects:

- We propose an enhanced (DRL) algorithm based on [9], which leverages low-cost, low-precision 2D LiDAR sensors for mapless navigation, enabling robust performance in complex environments without the need for high-precision maps.
- We propose a priority-based excellence experience collection mechanism and a multi-source experience fusion strategy. These strategies allow the agent to efficiently learn optimal policies from prior experiences, significantly reducing the training time required for effective navigation.
- Compared to other algorithms, our method can reach the destination quicker, safer, and more smoothly in unmapped and unknown environments, even when localization is inaccurate. Moreover, the strategies and hyperparameters in the simulation environment can be deployed in the real world without any adjustments.
- Additionally, our algorithm has been open-sourced, and the code repository is available at https://github.com/nfhe/darc_drl_nav (accessed on 28 August 2024).

The structure of this paper is as follows: Section 2 introduces related work. Section 3 focuses on the proposed algorithm. Section 4 presents the results of experiments, and the conclusions are provided in Section 5.

## 2. Related Works

Autonomous navigation and obstacle avoidance remain prominent research areas in mobile robotics [10,11,12,13]. Technological advancements have led to the emergence of more high-performance sensors and computing devices. This has markedly enhanced the accuracy and operability of real-time SLAM. Ranging sensors encompass RGB cameras [14,15,16], 2D lidar [17], 3D lidar [18], and multi-sensor fusion algorithms [19,20,21]. Despite advancements, most mobile robots depend on SLAM for indoor navigation [22,23,24]. This dependence presents a significant challenge for robots in unmapped and unknown environments.

The advancement of DRL algorithms, coupled with enhanced data processing capabilities, has broadened their application in autonomous navigation. One study [25] introduced an autonomous navigation and obstacle avoidance algorithm, merging the Artificial Potential Field (APF) method with DRL. This method adaptively optimizes the two-parameter gains of the APF controller through the actor–critic approach, efficiently completing the navigation task. Surmann et al. [26] developed a parallel acceleration algorithm that enhances learning strategies by integrating multi-sensor data fusion and relative positioning. Cimurs et al. [27] introduced a DRL-based framework for autonomous navigation and exploration of unknown environments. This framework utilizes potential navigation information to select optimal path points, addressing the local optimum problem in real-time planning. Enrico et al. [28] presented an algorithm based on Double Q-learning, employing parallel asynchronous training to expedite the learning of optimal control strategies. Lodel et al. [29] proposed a navigation strategy that maximizes information collection rewards based on reference trajectory points and integrates it with a Model Predictive Control (MPC) local planner. The robot’s control strategy is optimized by generating trajectories with enhanced safety constraints guided by reference points. Lee et al. [30] introduced a hierarchical reinforcement learning framework to streamline navigation strategy learning in agents. This approach minimizes the need for manual reward function selection and improves training convergence by using a broadly referenced collection reward instead of a fixed reward function. Murad et al. [31] addressed the issue of sparse reward functions using MPC. Their strategy efficiently resolves the sparse reward problem in navigation tasks, reducing the need for expert intervention and extensive parameter adjustments. Cui et al. [32] proposed an optimization algorithm using the control barrier function, enhancing the safety and flexibility of agents during navigation with lidar data. This algorithm leverages a large data model to perceive and predict the surrounding environment’s dynamics and the consequences of various agent behaviors. Shen et al. [33] presented a navigation framework combining mixed-weight trust region optimization with active learning for inverse reinforcement learning. This method integrates expert-level navigation data and domain knowledge to refine navigation strategies through weight learning. Xiong et al. [34] developed a navigation strategy that merges the twin neural Lyapunov function with a control strategy and DRL, focusing on safety in unknown, dynamic, and complex environments, an aspect often overlooked in traditional DRL strategies.

Although these strategies are effective in autonomous robot navigation, they exhibit limitations, including poor generalization, low learning efficiency, vulnerability to local optima, and challenges in transitioning from simulations to real-world applications. Consequently, we propose an enhanced DRL algorithm capable of completing navigation tasks autonomously, effective even in scenarios with mapless or low-accuracy mapping.

## 3. PEEMEEF-DARC Autonomous Navigation

To address mapless navigation in unmapped and unknown environments, we introduce a novel navigation framework, PEEMEEF-DARC. The framework consists of the following three key components: (1) an enhancement of the DDPG algorithm’s exploration capabilities, achieved by integrating an additional actor and a regularized critic to address low-value estimations in the critic; (2) a priority-based excellence experience collection, derived from the traditional navigation framework, which accelerates the agent’s learning of effective strategies; and (3) the integration of a multi-source experience fusion strategy module, further improving the agent’s learning efficiency and quality.

### 3.1. DARC Module

To enhance the autonomous navigation capabilities of robots in complex environments, we applied the DARC algorithm, as introduced in [9]. The core idea of the DARC algorithm is to reduce the bias in value estimation by employing two critics to estimate a weighted Q value. In traditional single-critic structures, the value estimation can often be overly optimistic or pessimistic, which negatively impacts the stability and effectiveness of policy learning. This bias is particularly problematic in complex and dynamic environments, where it can cause the robot to get stuck in local optima and fail to find the global optimum.

**Figure 3:**
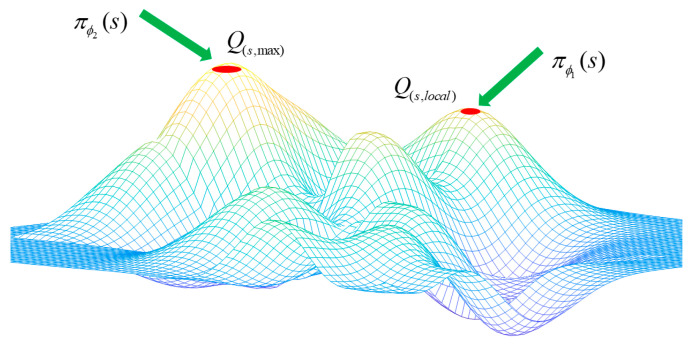
Double agents can assist in overcoming local optima.

Accurately estimating values is crucial for agents to acquire strategic understanding. Double actors allow agents to choose from multiple control strategies in diverse environments instead of being limited to a single condition. As depicted in Figure 3, the single-factor policy $\pi_{\phi_{1}}(s)$ could cause agents to settle for the local optimum $Q_{\left(s,local\right)}$, rather than the global optimum $Q_{\left(s,\max\right)}$, due to insufficient exploration performance.

To address these challenges, the DARC algorithm introduces a dual-critic structure, where each critic independently estimates the Q value for a given state–action pair. By averaging the estimates from both critics, the algorithm mitigates the bias that can result from relying on a single critic, leading to more accurate and reliable value estimations. This dual-critic approach not only enhances the robustness of the value estimation but also provides a broader perspective for policy evaluation, which is critical in navigating complex and uncertain environments. We start by initializing two critic networks $Q_{\theta_{1}}(s,a)$ and $Q_{\theta_{2}}(s,a)$, and two actor networks $a_{1}=\pi_{\phi_{1}}(s)$ and $a_{2}=\pi_{\phi_{2}}(s)$, where $\theta_{1},\theta_{2},\phi_{1}$ and $\phi_{2}$ represent the parameters of the respective networks. During each training step, both critic networks are used to estimate the Q value [35]:

(1) $$Q(s,a;\theta^{Q})=min(Q_{\theta_{1}}(s,a_{1}),Q_{\theta_{2}}(s,a_{2}))$$

This approach is intended to provide a more conservative estimate by selecting the lower of the two Q-values, which may help avoid the risks associated with overestimation in policy updates.

Subsequently, we construct the critic network parameters $\theta_{1},\theta_{2}$ on the double actors. To select the value function, the DARC algorithm employs a conservative approach by first selecting the minimum Q value estimated by the critic network for each strategy $\pi_{\phi_{i}}\left(s^{\prime }\right),i=1,2$. This helps in reducing the risk of overestimation in policy updates. Then, among these minimum Q values, the algorithm selects the maximum value, ensuring a balanced estimation across different strategies and enhancing the stability of the learning process. Unlike traditional Double Q learning algorithms, it employs a value function for calibrating value estimation instead of independently updating the estimated target value through the actor–critic.

To enhance the accuracy of these estimates, DARC integrates a regularization mechanism that penalizes significant deviations between the two critics’ estimates. The regularized loss function for the critics is defined as:

(2) $$L(\theta )=E_{\left(s,a\sim D\right)}{\left[r+\gamma \underset{i=1,2}{\min}Q_{{\theta^{\prime }}_{i}}(s^{\prime },\pi_{\theta^{\prime }}(s^{\prime }))-Q_{\theta }(s,a)\right)}^{2}]+\lambda {\left(Q_{\left(\theta_{1}\right)}(s,a)-Q_{\left(\theta_{2}\right)}(s,a)\right)}^{2}$$

where r denotes reward, γ is the discount factor, and $Q_{{\theta^{\prime }}_{i}}(s^{\prime },\pi_{\theta^{\prime }}(s^{\prime }))$ represents the target Q-value. The regularization term $\lambda {\left(Q_{\left(\theta_{1}\right)}(s,a)-Q_{\left(\theta_{2}\right)}(s,a)\right)}^{2}$ ensures that the estimates from the two critics remain close, thereby preventing large discrepancies that could destabilize the learning process.

Additionally, to facilitate a smoother convergence of the policy, a soft update mechanism is employed to combine the critics’ value estimates:

(3) $$\hat{Q}(s,a)=\upsilon \min\left(Q_{1}\left(s,a;\theta_{1}\right),Q_{2}\left(s,a;\theta_{2}\right)\right)+(1-\upsilon )\max\left(Q_{1}\left(s,a;\theta_{1}\right),Q_{2}\left(s,a;\theta_{2}\right)\right)$$

Here, υ serves as a parameter that adjusts the weight given to each Q-value estimation path. When υ approaches 0, the algorithm leans towards a more conservative estimate, minimizing potential overestimation. Conversely, as υ approaches 1, the algorithm becomes more optimistic in its value estimates. This mechanism allows the DARC algorithm to dynamically balance the exploration–exploitation trade-off during training, thereby improving the agent’s learning efficiency and robustness.

The DARC algorithm also leverages the interaction between the actor and critic networks to enhance policy learning. The policy is updated based on the critic’s evaluations, where the objective is to maximize the expected return:

(4) $$J(\phi )=E_{s∼D}\left[Q_{\theta }\left(s,\pi_{\phi }(s)\right)\right]$$

To optimize this objective, we perform gradient ascent on the actor’s parameters:

(5) $$\nabla_{\phi }J(\phi )=E_{s∼D}\left[\nabla_{\phi }Q_{\theta }\left(s,\pi_{\phi }(s)\right)\right]$$

Given that the policy’s effectiveness is directly influenced by the accuracy of the Q value estimates, the DARC algorithm’s dual-critic structure plays a crucial role in ensuring that these estimates are reliable, thereby stabilizing the entire learning process. Furthermore, the regularization applied to the critics not only reduces the underestimation bias but also ensures that the estimates remain consistent across different states and actions. This consistency is essential for maintaining the stability of the policy learning process, especially in environments where the state–action space is large and highly variable.

To further stabilize learning, DARC employs target networks for both the actors and critics. The target networks $\theta_{1}^{\prime },\theta_{2}^{\prime }$ and $\phi_{1}^{\prime },\phi_{2}^{\prime }$ are periodically updated with the parameters of the respective primary networks, ensuring that the target values used in updates do not change too rapidly. The target Q values are computed as:

(6) $$y=r+\gamma \underset{i=1,2}{\min}Q_{\theta_{i}^{\prime }}\left(s^{\prime },\pi_{\phi_{i}^{\prime }}\left(s^{\prime }\right)\right)$$

The temporal difference error, which drives the learning process, is then calculated as:

(7) $$\delta =y-Q_{\theta }(s,a)$$

By minimizing this temporal difference error, the critic networks can iteratively refine their Q-value estimates, potentially improving the accuracy of the value function over time.

The DARC algorithm, through its use of double critics, regularization, and soft updates, offers a structured approach to addressing the challenges of autonomous navigation. These mechanisms are intended to provide a more stable and accurate framework for policy learning, which could be particularly beneficial in complex and dynamic environments.

### 3.2. Priority Excellent Experience Date Collection Module

In this study, we introduced the use of the ROS move base package to facilitate automated data collection. Traditional experience collection methods often rely on manual configurations and predefined strategies. While these methods can be effective in controlled environments, they present several notable limitations when applied in real-world scenarios. Firstly, manual configurations are inherently subjective, leading to data that may lack the flexibility and adaptability required in complex and unknown environments. The fixed nature of these configurations often restricts the range of exploration, resulting in a dataset that may not be sufficiently diverse. This limitation can hinder the generalization of learned strategies, making them less effective when applied to a broader range of conditions. Moreover, manual configurations are prone to human bias, which can affect the objectivity of the collected data and ultimately reduce the effectiveness of the autonomous navigation tasks.

**Figure 4:**
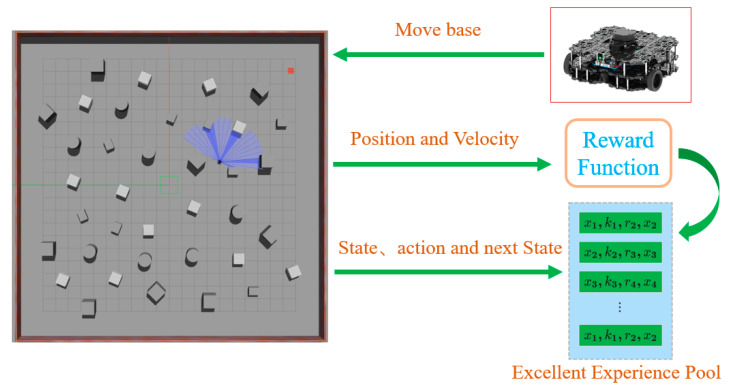
Priority excellent experience data collection strategy.

To overcome these limitations, we employed the move base package to enable autonomous data collection. As illustrated in Figure 4, the robot can autonomously navigate through various environments and collect critical navigation data in real time, including linear $l_{t}$ and angular velocities $v_{t}$, laser scan data $l_{t}$, the relative positions of goals $p_{t}$, and the reward function value $r_{t}$. This state $S_{t}$ is crucial for determining the appropriate action $a_{t}$, which the robot will execute based on the current state.

This automated data collection approach not only minimizes human bias but also enhances the diversity of the dataset, ensuring that the collected data are comprehensive and representative of the operational environment. The actions $a_{t}$, derived from state $S_{t}$, lead to transitions to the next state $S_{t+1}$, while a reward $r_{t}$ evaluates the effectiveness of the action. This interaction between states, actions, and rewards, as shown in Figure 4, forms the basis of the learning process, allowing the robot to refine its strategies over time.

Additionally, the move base package ensures that the data collection process is seamlessly integrated with the navigation tasks, thereby improving the relevance and effectiveness of the collected data. Compared to traditional methods, this autonomous approach provides a robust foundation for subsequent learning and strategy optimization, ultimately shortening training times and improving the generalization capabilities of the strategies.

All collected data are stored in a priority-based excellence experience pool. This experience pool allows the agent to prioritize learning from high-quality strategies, thereby enhancing training efficiency and better preparing the agent for autonomous navigation in unknown environments.

### 3.3. Multi-Source Experience Fusion Strategy Module

In this module, we propose a multi-source experience fusion strategy based on a reward value function. This strategy integrates prioritized excellent experience data into an excellent experience pool and combines this with ordinary experiences according to a predefined ratio during learning. This approach enables the agent to rapidly learn desired actions, thus efficiently speeding up the training process. Figure 2 illustrates the experience pool positioned at the framework’s core. Its primary function is to store the agent’s current actions, sensor data generated by environmental changes, and corresponding reward values. The implementation steps of this algorithm are as follows:

(1) Real-time sorting of the data in the experience pool according to the reward value $r_{t}$, and the data with the top P% reward values are divided into the excellent experience pool. In the process of data selection, samples are prioritized from this excellent experience pool.
(2) In each iteration, the agent generates new data $s_{t}$. If the reward value in this data exceeds the set excellent experience pool threshold, it is added to this pool.
(3) For each training session, a total of A data sets are chosen. A∗M% are randomly selected from the excellent experience pool and A∗(1−M%) from the ordinary pool. These are then randomly combined for training.
(4) After training, the agent blends the excellent with the ordinary experience pool. The excellent pool and its reward threshold are reset based on the top P% in the experience pool.

**Figure 5:**
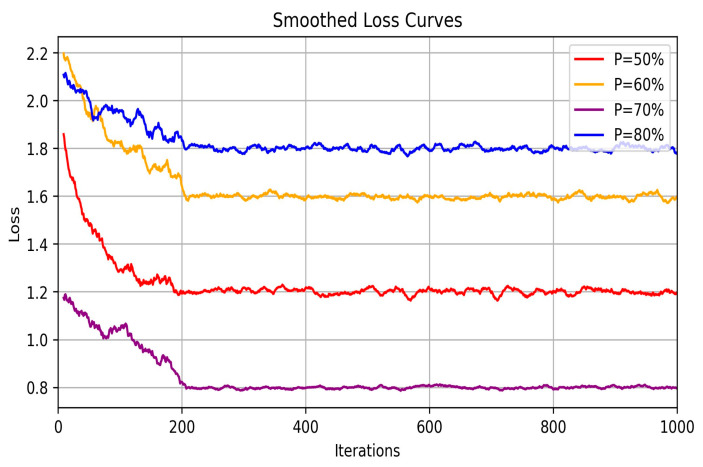
Ablation study of multi-source experience fusion strategy.

In this study, the term “multi-source” experience does not refer to data from multiple simulation environments but rather to the variety of experiences generated within a single simulation environment through iterative training. These experiences reflect the different scenarios and outcomes encountered by the agent during task execution. By integrating and sorting these diverse experience data based on reward values, the agent can more effectively identify and learn optimal strategies. To further evaluate the impact of different proportions of prioritized experience in the multi-source experience fusion strategy, we conducted ablation experiments. Specifically, we selected various proportions of prioritized experience (*p*-values of 50%, 60%, 70%, and 80%) for comparative analysis, aiming to assess how these proportions influence the convergence rate and final stability of the loss function during training. The experimental results, as shown in Figure 5, reveal the convergence trends of the loss function under different *p*-values. Analyzing these experimental data, we observe that as the proportion of prioritized experience increases, the algorithm’s convergence rate significantly improves. Notably, at *p* = 70%, the loss value reaches its lowest point, indicating that the model exhibits the best learning performance under this condition, with the most stable and smooth convergence of the loss function. Specifically, when the *p*-value is 50%, the loss function, although it decreases rapidly in the initial stages, exhibits considerable volatility in the later stages of training, resulting in poor convergence stability. In contrast, when the *p*-value is 70%, the loss function not only decreases rapidly in the initial stages but also shows the most stable convergence trend in the later stages. This suggests that the strategy provides the best support for model training at this proportion, reducing volatility during the training process.

The design of this multi-source experience fusion strategy enables the agent to learn the desired actions from better strategies. Especially in the early stages of training, this strategy helps the agent to learn more effectively, quickly escape from local optima, and accelerate the convergence process of the algorithm, thereby improving the efficiency and quality of the entire training.

### 3.4. Reinforcement Learning Setting Module

(1) State Space and Action Space

State space: $s_{t}=\{l_{t},v_{t},\omega_{t},p_{t}\}$, where $l_{t}$ represents 2D lidar data, $v_{t}$ denotes the linear velocity of agents, $\omega_{t}$ stands for the angular velocity of agents, and $p_{t}$ denotes the relative position of the goal. Action space: $a_{t}=\{l_{v},a_{v}\}$, where the neural network provides the linear velocity $l_{v}$ and the angular velocity $a_{v}$.

(2) Reward Function Design

The goal of agent training is to achieve autonomous navigation in an unmapped and unknown environment. The agent aims to reach the goal in the shortest time, on the safest path, and with a smooth trajectory. To support this objective, we are developing a new reward function:

(8) $$r=(r_{g}+r_{w}+r_{c}+r_{o}+r_{v})$$

We hope that the agent moves towards the goal g. $P_{t}$ and $P_{t+1}$ represent the positions of agents at two successive time intervals, respectively. To quantify our approach, we set a constant value $C_{g}=0.6$. The reward $r_{g}$ is defined as follows:

(9) $$r_{g}=\left\{\begin{array}{cc} 400 & ||p-g||<0.5\\ C_{g}\left(\left||p_{t+1}-g||-||p_{t}-g|\right|\right) & otherwise \end{array}\right.$$

We designed a reward function for angular velocity. If the angular velocity surpasses a specific limit, a significant penalty is applied. The reward $r_{w}$ is represented as follows:

(10) $$r_{w}=\left\{\begin{array}{cc} -0.5 & Go\;straight\;and\;|\omega |<0.1\\ -2 & \;0.1<|\omega |<0.6\\ -100 & \;|\omega |>0.6\\ 0 & otherwise \end{array}\right.$$

If the agent is close to an obstacle or collides with it, a penalty is applied. We set $d_{\min}$ as the distance between the agent and the obstacle, establish the collision threshold at $d_{n\min}$, and assign $C_{c}=20$. The expression is as follows:

(11) $$r_{c}=\left\{\begin{array}{cc} C_{c}(d_{\min}-0.2) & \;d_{n\min}<d_{\min}<2\times d_{n\min}\\ -200 & \;d_{\min}<d_{n\min}\\ 0 & otherwise \end{array}\right.$$

To improve the flexibility and smoothness of agents, a penalty is applied when oscillations occur.

(12) $$r_{o}=\left\{\begin{array}{cc} -5|\Delta \omega_{t}| & |\Delta \omega_{t}|>0.6\\ 0 & otherwise \end{array}\right.$$

To accelerate the agent’s arrival, we set $C_{v}=10$. The reward $r_{v}$ is defined as follows:

(13) $$r_{v}=\left\{\begin{array}{cc} C_{v}\times v_{t} & \;\left|v_{t}\right|>0.2\;\\ -2 & \;\left|v_{t}\right|<0.2\\ 0 & otherwise \end{array}\right.$$

(3) Network Structure

**Figure 6:**
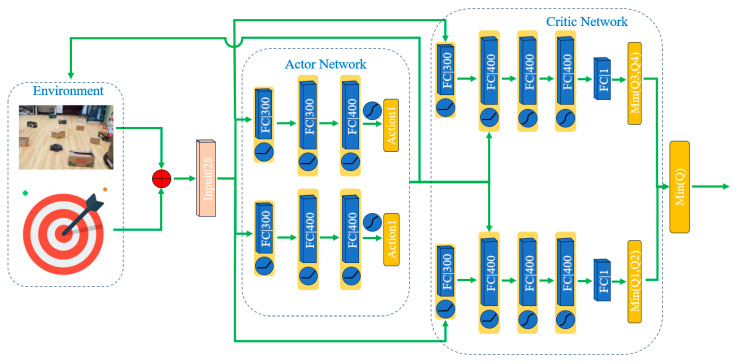
The DRL algorithm comprises actors and critics, with each layer specifying its corresponding number of parameters.

The DARC algorithm comprises three components, actor, critic, and their corresponding target networks, as illustrated in Figure 6. These target networks are structurally identical to the original networks. The actor network is composed of an input layer, three hidden layers, and an output layer. The input layer receives a 28-dimensional vector, which includes a 24-dimensional vector from the lidar, the distance to the goal, the heading angle deviation, and the agent’s linear and angular velocities. The hidden layers contain 300, 400, and 400 neurons, respectively, each employing a rectified linear unit (ReLU) for activation. The output layer utilizes the hyperbolic tangent function (Tanh) to determine the action space. This output from the action space is then fed into the critic network as input. The structure of the critic network closely mirrors that of the actor, with four hidden layers: the first containing 300 neurons and the remaining three, 400 each. All hidden layers in the critic network employ ReLU activation functions to compute the final Q value. Differing from the actor network, the critic’s input layer merges with the actor’s output after the first hidden layer and then continues through the second, third, and fourth hidden layers. This architecture enables the DARC algorithm to effectively handle complex decision-making tasks, particularly in scenarios involving high-dimensional input data and the requirement for precise outputs.

## 4. Experiments

### 4.1. Environment and Parameter Settings

**Figure 7:**
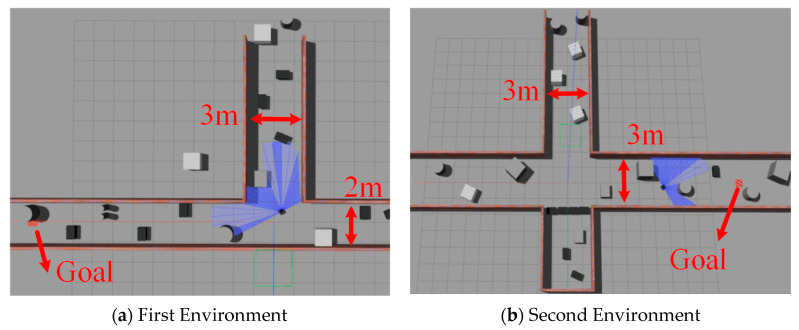
Unmapped and unknown environment employed for the training of the robot.

Training the algorithm using Gazebo reduces reliance on environmental information. Additionally, the consistency of the robot model in the simulation with its real-world counterpart aids in a smoother transition from simulation to real-world application. To enhance the network’s generalization and robustness, Gaussian noise is added to sensor data and action values during training to improve the network’s generalization and robustness. First, to demonstrate the effectiveness of the improved algorithm, we conducted ablation experiments in unknown environments, as depicted in Figure 4. These ablation studies specifically analyzed the impact of each component of the PEEMEF-DARC framework, including the double actors regularized critics, the priority-based excellence experience data collection mechanism, and the multi-source experience fusion strategy mechanism. In the multi-source experience fusion strategy, the results of these experiments confirmed the significant contribution of each component to the overall performance of the algorithm, with particular emphasis on how varying the proportion of prioritized experiences affected the learning process. Second, the challenges of localization and stability in unmapped environments present a rigorous test for autonomous navigation systems. Evaluating the algorithm’s performance in these environments offers a stringent assessment of its robustness and generalization capabilities. Therefore, we performed a comprehensive comparison of our algorithm with other baseline methods in unmapped environments, as shown in Figure 7. In these experiments, the robots start at the position [0,0], with goals randomly generated and marked by red squares. The 24 sparse data points from the 2D lidar, depicted as blue laser lines, provide critical sensor information. This sensor has a 180° Field Of View (FOV) and a scanning range of 0.12 m to 3.5 m. These additional tests, including the variation in prioritized experience ratios, not only reinforce the algorithm’s versatility across various scenarios but also highlight its capacity to perform effectively in challenging environments where traditional SLAM-based methods might struggle with map inaccuracies.

For quantitative experiments and to enable comparisons with computationally constrained algorithms, the agent training was conducted on a computer system equipped with an NVIDIA GTX 2060 graphics card, 32 GB of memory, and an AMD R7 CPU. The actor–critic networks utilized an Adam optimizer with respective learning rates of 0.0001 and 0.001. The soft update factor is set to 0.001, and the regularization coefficient to 0.01. The threshold for advancing to the next round was determined by the performance outcomes of the preceding round. The buffer size is $2\times 10^{6}$, comprising an excellent experience pool and an ordinary experience pool, each of size $10^{6}$. The coefficient for the excellent experience pool is denoted by P=70%. The batch factor is set at 256. Training terminates under any of the following four conditions: (1) the agent reaches the goal; (2) collision between the agent and obstacles; (3) cumulative training steps reach 1000; (4) reward for a single training session exceeds −30.

To showcase the proposed algorithm’s superior performance, we conducted comparative experiments with various algorithms, including DDPG, DA-DDPG, PPO [36], HYC-DDPG [37], GD [27], and CROP [38]. Notably, PPO is recognized by DeepMind as an important benchmark in assessing reinforcement learning algorithm performance. Additionally, three state-of-the-art autonomous navigation algorithms—HYC-DDPG, GD, and CROP—were selected for an in-depth comparative analysis.

### 4.2. Comparison of Experimental Results

In this study, we initially recorded 2000 sets of prioritized excellent experience data from the environment depicted in Figure 4. Subsequently, we conducted detailed ablation studies to verify the specific contributions of the improved DRL algorithm to performance. The aim of these studies was to analyze how each component of the algorithm affects overall performance, thereby deeply understanding the improved algorithm’s effectiveness.

(1) Results of ablation experiment

**Figure 8:**
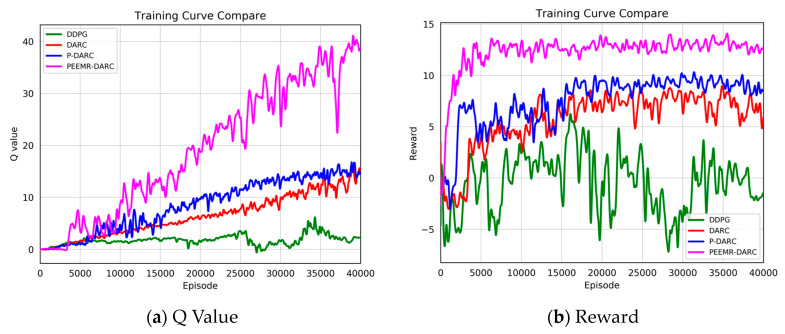
Ablation study of an enhanced DRL algorithm in unmapped and unknown environments.

To demonstrate the advantages of our improved DRL algorithm in autonomous navigation in unknown environments, experiments were conducted in the complex environment shown in Figure 4. Results in Figure 8 indicate that the improved DARC algorithm significantly outperforms the original DDPG algorithm. Additionally, the prioritized excellent experience strategy notably accelerates the algorithm’s convergence speed. Most notably, the multi-source experience fusion strategy module was observed to be crucial in enhancing the performance of autonomous navigation algorithms, particularly in terms of robustness and adaptability in diverse environments.

(2) Simulation Environment Training

**Figure 9:**
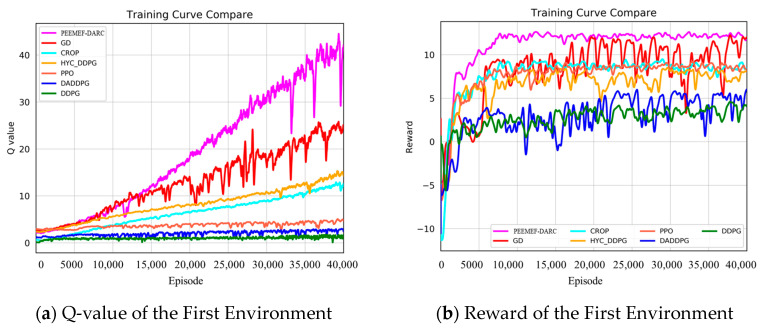
Q value and reward value in the first environment.

**Figure 10:**
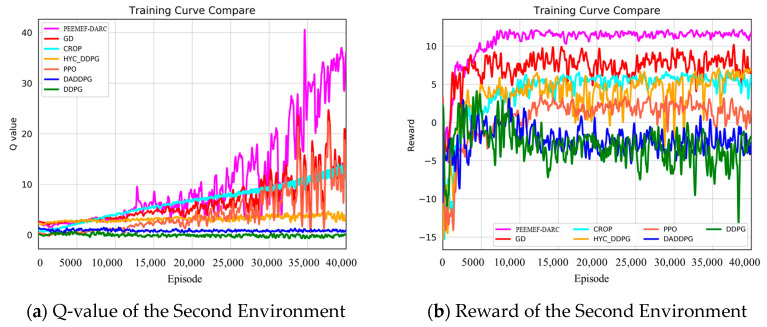
Q value and reward value in the second environment.

Figure 9 and Figure 10 present a comparison of training outcomes in two different unknown environments.

To mitigate the effect of randomness on experimental outcomes, we averaged the Q-values and reward values over n consecutive steps. In a 3 m wide, unmapped, and unknown simulated environment, our algorithm demonstrated faster and more stable increases in Q-values and reward values. Notably, at 15,000 training steps, the Q-value of our algorithm significantly surpassed those of other algorithms. For instance, the Q-values for the GD, CROP, and HYC-DDPG algorithms were approximately 20, 14, and 11, respectively, compared to approximately 5, 3, and 1 for PPO, DA-DDPG, DDPG, and others. Similarly, the trend in reward values paralleled that of the Q-values, stabilizing at around 8000 steps and maintaining a level of about 15, whereas other algorithms’ rewards were around 7, 5, 5, 2, −2, and −3, respectively. In a more complex 2 m wide simulated environment, our algorithm consistently and rapidly attained higher Q-values and reward values. The heightened complexity of this environment increased the difficulty for the agent to navigate, resulting in generally lower Q-values and reward values for all algorithms. As Figure 10 illustrates, our algorithm’s Q-value reached 10 at 24,000 steps and continued to rise throughout subsequent training. The highest Q-value achieved by other algorithms at 40,000 steps was 25, markedly lower than that of our algorithm at the same stage. The reward values trended similarly to the Q-values, stabilizing after 10,000 training steps and remaining around 12 over the long term. These results demonstrate that our algorithm attains higher Q-values and reward values in unknown, complex, narrow environments, significantly shortening training duration and exhibiting superior performance.

(3) Simulation Environment Results

**Figure 11:**
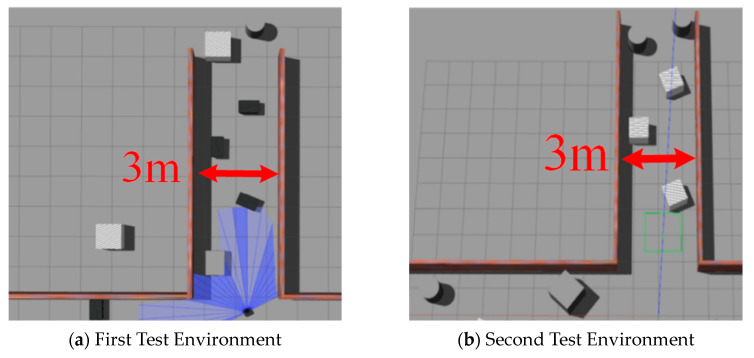
The unmapped environment used for robot testing.

To more comprehensively assess the adaptability of the algorithm, we conducted additional tests in two newly designed simulation environments. Figure 11a,b illustrates the configurations of these new test environments. These environments were specifically designed with varying obstacle positions to further challenge the algorithm’s ability to handle diverse scenarios. Through testing these environments, our aim is to evaluate the robustness and adaptability of the algorithm when faced with varying environmental conditions. These additional tests are intended to provide a more thorough evaluation of the algorithm’s performance in complex and unfamiliar settings.

**Figure 12:**
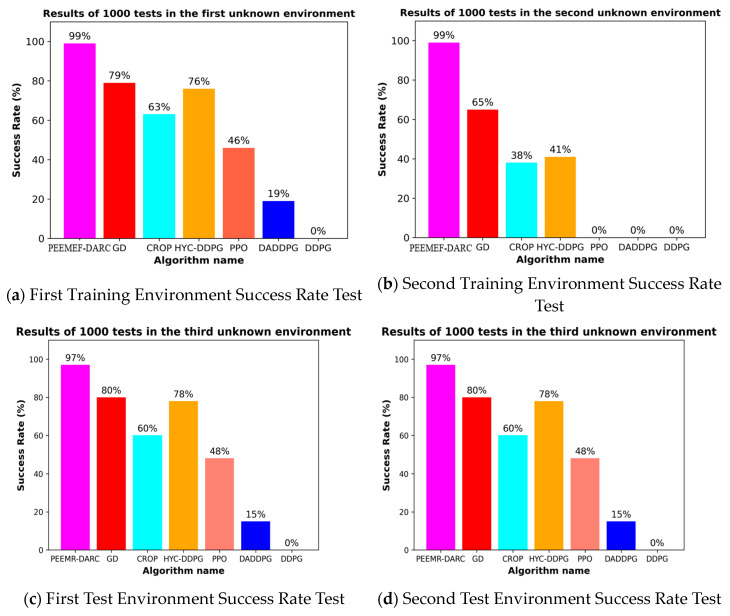
Success rate of 1000 tests in unmapped and unknown environments.

Upon completion of the initial training in two environments, we conducted 1000 random task tests to evaluate the trained models’ performance. To further validate the robustness and adaptability of the proposed algorithm, two additional testing environments were introduced, as shown in Figure 12c,d. These additional environments were designed to assess the algorithm’s generalization capability beyond the training scenarios. The results indicate that the success rates in the newly added environments were consistent with the trends observed in the initial training environments, demonstrating the effectiveness of the improved DRL algorithm in navigating unmapped and unknown environments. Moreover, this extended testing confirms that the proposed method maintains a higher success rate compared to other algorithms across different environments.

The experimental results are summarized in Table 1. Across all four unmapped and unknown environments, the proposed PEEMEF-DARC algorithm consistently achieves the shortest average path length among the compared algorithms, particularly in Environment-1 and Environment-2. Even in the newly introduced Environment-3 and Environment-4, where the complexity was increased, our algorithm continued to demonstrate its efficiency by producing shorter average paths. This outcome indicates that the PEEMEF-DARC algorithm, while maintaining high success rates, also excels in real-time decision-making, effectively minimizing the total distance traveled by the robot from the start to the destination. The algorithm’s ability to generate efficient paths in real time underscores its robustness and adaptability across varying environmental conditions, making it a highly effective solution for autonomous navigation in complex settings.

(4) Real–World Environment Results

**Figure 13:**
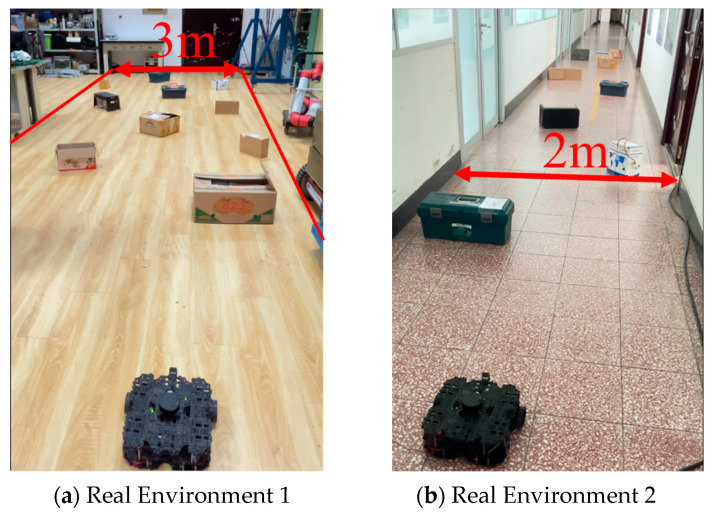
Unknown and complex experimental environments in real world.

**Figure 14:**
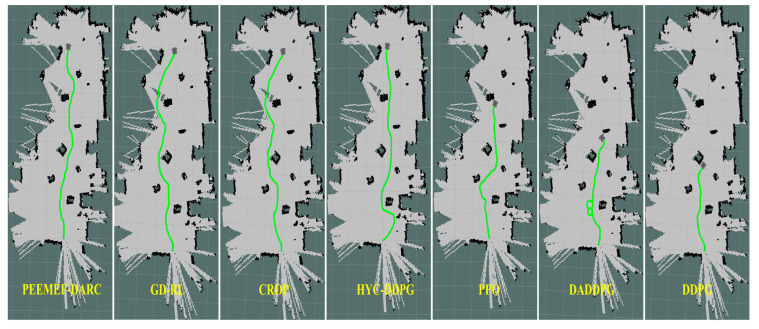
Comparison of trajectories in the first environment.

**Figure 15:**
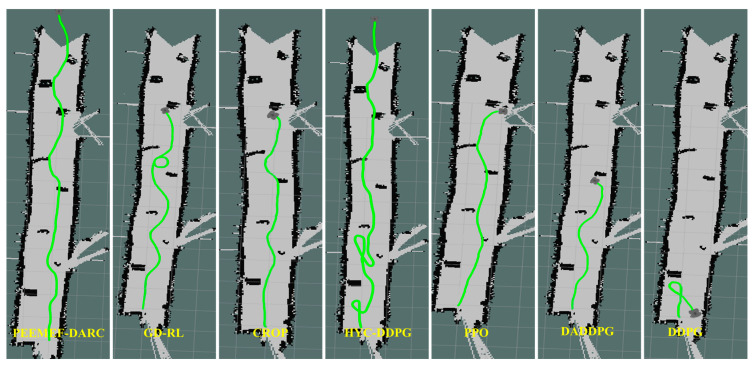
Comparison of trajectories in the second environment.

To further verify its applicability and effectiveness, the model was implemented in an actual robot for real-world testing. Tests were conducted in the laboratory and corridors of Block R at the Shenyang Institute of Automation, Chinese Academy of Sciences, featuring two randomly created unmapped and unknown environments, as shown in Figure 13. In these real-world experiments, the agent’s position was estimated using the AMCL algorithm. Figure 14 and Figure 15 display the robot’s trajectory in the actual environment. In these real-world experiments, the robot’s position was estimated using the Adaptive Monte Carlo Localization (AMCL) algorithm. However, it was observed that in Environment 2, the AMCL algorithm struggled with accurate positioning due to the long, narrow corridor, leading to occasional trajectory intersections with obstacles. Despite these challenges, our proposed PEEMEF-DARC algorithm demonstrated robust performance, effectively addressing the positioning inaccuracies and maintaining a high success rate.

**Figure 16:**
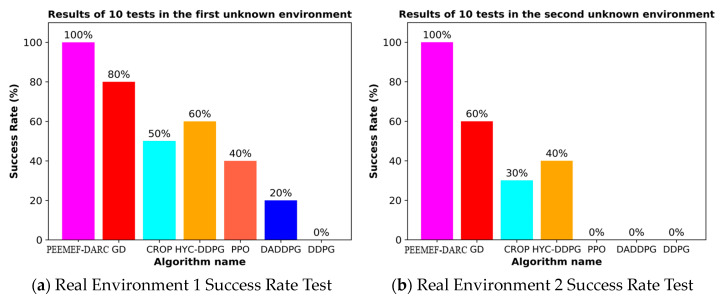
Success rate of 10 tests in two environments.

To thoroughly evaluate our algorithm’s performance in autonomous navigation within unmapped and unknown environments, we executed 10 sets of repetitive tests. These tests specifically examined the agent’s navigation capability under challenging conditions, including the absence of prior map knowledge, incomplete map information, or inaccurate positioning. The experimental results, as depicted in Figure 16, demonstrate that our algorithm can make effective decisions under these challenging conditions, even while relying on low-precision sensors. This capability enables the agent to operate in a broader range of environments, transcending the limitations of known maps and precise positioning systems. Notably, our algorithm excelled in all test tasks, in contrast to comparative algorithms that failed to successfully complete them. This outcome underscores our algorithm’s substantial advantages in navigating unknown complex environments. These experiments show the efficiency, adaptability, and robustness of our algorithm, particularly in unmapped and unknown environments.

Our experimental results are presented in Table 2. Under the condition of maintaining high success rates, the proposed algorithm achieves the shortest average path length in both real-world environments. This demonstrates that our algorithm not only guarantees high success rates but also significantly enhances path planning efficiency, effectively minimizing the total distance traveled by the robot from the start to the destination.

## 5. Conclusions

This study successfully developed an autonomous navigation and obstacle avoidance algorithm for mobile robots tailored for unmapped and unknown environments. Our enhanced DRL algorithm significantly improves exploration capacity, strategy learning speed, and overall learning efficiency in mobile robots compared to other algorithms. The implementation of regularization effectively addresses the overestimation problem often encountered in action-value predictions across varying states. Furthermore, the priority excellent experience collection and the multi-source experience fusion strategy developed in this study, significantly shorten the learning duration, allowing the agent to rapidly acquire effective strategies. Experimental results indicate that incorporating these strategic modules can reduce training time by approximately 60%. Our algorithm consistently shows superior performance in unmapped and unknown environments, particularly in scenarios such as narrow corridors approximately 2 m wide, compared to other DRL algorithms tested. In the future, we aim to apply our algorithm to indoor unmanned inspections, enhancing the autonomous decision-making capabilities of robots. Concurrently, we plan to further optimize the architecture to enable robots to predict the behavior of dynamic obstacles using multi-sensor fusion algorithms.

## Figures and Tables

**Table 1:** Average length (m) of 1000 tests in four unmapped and unknown environments.

Environment	PEEMEF-DARC	GD	CROP	HYC-DDPG	PPO	DADDPG	DDPG
Environment-1	12.32	13.02	12.68	13.59	12.86	14.65	-
Environment-2	13.56	13.95	14.14	14.87	-	-	-
Environment-3	12.93	13.35	13.31	14.05	13.50	15.38	-
Environment-4	13.24	14.05	14.01	15.11	-	-	-

**Table 2:** Average length (m) of 10 tests in two unmapped and unknown environments.

Environment	PEEMEF-DARC	GD	CROP	HYC-DDPG	PPO	DADDPG	DDPG
Environment-1	8.54	9.02	8.86	9.25	8.95	9.85	-
Environment-2	15.68	15.89	16.62	16.54	-	-	-

## Data Availability

https://github.com/nfhe/darc_drl_nav (accessed on 29 August 2024).
